# Comparing Radar-Based Breast Imaging Algorithm Performance with Realistic Patient-Specific Permittivity Estimation

**DOI:** 10.3390/jimaging5110087

**Published:** 2019-11-19

**Authors:** Declan O’Loughlin, Bárbara L. Oliveira, Martin Glavin, Edward Jones, Martin O’Halloran

**Affiliations:** Electrical and Electronic Engineering, National University of Ireland Galway, Galway H91CF50, Ireland; barbara.oliveira@nuigalway.ie (B.L.O.); martin.glavin@nuigalway.ie (M.G.); edward.jones@nuigalway.ie (E.J.); martin.ohalloran@nuigalway.ie (M.O.)

**Keywords:** radar-based breast imaging, microwave imaging, breast cancer

## Abstract

Radar-based breast imaging has shown promise as an imaging modality for early-stage cancer detection, and clinical investigations of two commercial imaging systems are ongoing. Many imaging algorithms have been proposed, which seek to improve the quality of the reconstructed microwave image to enhance the potential clinical decision. However, in many cases, the radar-based imaging algorithms have only been tested in limited numerical or experimental test cases or with simplifying assumptions such as using one estimate of permittivity for all patient test cases. In this work, the potential impact of patient-specific permittivity estimation on algorithm comparison is highlighted using representative experimental breast phantoms. In particular, the case studies presented help show that the permittivity estimate can impact the conclusions of the algorithm comparison. Overall, this work suggests that it is important that imaging algorithm comparisons use realistic test cases with and without breast abnormalities and with reconstruction permittivity estimation.

## 1. Introduction

Radar-based imaging is an emerging modality for breast cancer detection [1]. In recent years, a number of clinical studies have been published [2,3,4,5,6,7], including the commercially available MARIA^®^ system (Micrima Ltd., Bristol, UK). A competing system developed by Microwave Vision Group (Villebon-sur-Yvette, France) is being used in clinical investigations at the National University of Ireland Galway (Ireland) [8,9,10]. The increasing amount of clinical studies of microwave breast imaging systems is motivating further research into the optimal imaging algorithms and system design which can maximize the clinical efficacy of radar-based breast imaging [11].

Several imaging reconstruction algorithms for radar-based imaging have been developed (known as beamformers) and many introductory books, comprehensive reviews and open-source implementations of these algorithms have been published [12,13,14,15]. These beamformers have also been compared using a variety of test cases with both numerical and experimental breast phantoms and patient images [16,17,18,19,20,21,22]. However, many comparative studies to date have used a limited set of beamformers [16], simplified numerical models [17,20], idealized artefact removal algorithms [18], or considered only test situations with abnormalities [21]. A more exhaustive comparative study using five clinical case studies identified that the Delay-Multiply-and-Sum (DMAS) algorithm achieved the highest signal-to-clutter ratios (SCRs) for the five clinical case studies, but did not consider the potential impact of variations in breast dielectric properties between the five patients [22].

However, it is known that an incorrect estimate of the breast dielectric properties can impair the image quality of radar-based breast images [23]. In particular, assuming the same estimate of breast dielectric properties for each test case has been shown to impair the sensitivity of radar-based breast imaging where tumors are not correctly detected [24]. Several methods to identify the optimal patient-specific dielectric properties have been proposed [25,26], many of which rely on the properties of the reconstructed image. Similarly, some preliminary studies have suggested that DMAS beamformer may “improve” the image quality in cases where no abnormalities exist [22,27]. However, to date, no study has analyzed the impact of different reconstruction algorithms on patient-specific permittivity estimation algorithms, nor has the potential benefit of improved imaging algorithms been examined with patient-specific estimation algorithms.

The goal of this study is to examine the impact of patient-specific permittivity estimation algorithms on beamformer comparisons in healthy and tumor experimental models. Although patient-specific permittivity estimation and sophisticated beamformers have been shown to improve image quality independently in certain cases, the impact of patient-specific permittivity estimation on beamformer comparative studies is unknown. The remainder of this paper is structured as follows: Section 2 reviews the beamformers which have been proposed in the literature as well as patient-specific permittivity estimation algorithms. Section 3 describes the experimental set-up used to test the algorithms. The results are presented in Section 4 and, finally, Section 5 concludes the paper.

## 2. Background

Many reviews, books and editorials have been published about microwave imaging and microwave breast imaging in recent years [1,11,12,13,14,15,28,29,30,31,32,33,34,35,36,37] which describe the mathematical background and existing literature of the method. Broadly speaking, microwave breast imaging algorithms attempt to infer information about the dielectric contrast of the breast, and correspondingly, information about any tumors or other abnormalities present. The subject of this work is radar-based imaging, a qualitative technique which reconstructs an energy map of the backscattered energy within the breast. As tumor tissues often have the highest dielectric properties [1], large reflections occur at tumor tissue boundaries. In areas of high dielectric contrast (i.e., tumors), these reflections cohere and result in high image energy, whereas in areas of lower contrast, these reflections do not cohere and result in lower image energy.

The Delay-and-Sum (DAS) imaging algorithm was the first proposed for radar-based breast imaging and has been used in both mono- and multistatic configurations [38,39]. Mathematically, DAS can be represented as follows:

(1) $$I\left(r\right)=w\left(r\right)\int_{\Omega }\;P\left(\omega \right)\;\int_{A}\;\;\int_{A^{\prime }}w_{a,a^{\prime }}\left(r\right)E_{a,\omega }\left(a^{\prime }\right)\exp\left[j\omega \tau_{a,a^{\prime }}\left(r,\omega \right)\right]\;da\;da^{\prime }\;d\omega$$

where the image intensity, I(r), at the point r is calculated from the scattered energy, $E_{a,\omega }\left(a^{\prime }\right)$, recorded at antenna $a^{\prime }\in A^{\prime }$ while the incident signal, P(ω), is transmitted from antenna a∈A at angular frequency ω∈Ω. The propagation time for a signal from the transmitting antenna, a, to the point of interest, r, and back to the recording antenna, $a^{\prime }$, is represented by $\tau_{a,a^{\prime }}\left(r,\omega \right)$. DAS has been used in most patient imaging studies to date [3,4,5,40], and most comparative studies on imaging algorithm performance have used numerical or experimental data only [1,22].

Many extensions to DAS have been proposed which aim to improve the image quality. The extensions can be broadly classified into three types:

1. calculating a separate weighting factor to reward points where tumors are more likely based either on epidemiological studies or on characteristics of the scattered signals (w(r) from Equation (1));
2. prioritizing scattered signals collected at certain locations based on the relative locations of the antennas and points of interest or on the antenna radiation patterns ($w_{a,a^{\prime }}\left(r\right)$ in Equation (1));
3. by improving the quality of the input signals prior to imaging through improved artefact removal algorithms or other noise reduction techniques.

In most cases, these modified beamformers have been tested in limited numerical or experimental test cases, and most radar-based operational systems have used traditional DAS [1]. In this work, the DAS beamformer is compared to the Delay-Multiply-and-Sum (DMAS) beamformer which has been identified as improving image quality in a series of clinical case studies [22]. In the literature, DMAS is increasingly being used in new experimental studies without comparison to DAS or evaluation in imaging scenarios without targets [41,42].

The DMAS beamformer was first proposed in [43]. DMAS aims to reduce noise in the scattered signals and hence, reduce clutter in the image. After synthetic focusing and prior to summation in Equation (1), the scattered signals are pairwise multiplied, such that:

(2) $$I\left(r\right)=\int_{\Omega }\;P\left(\omega \right)\;\int_{A}\;\;\int_{A^{\prime }}\;\;\int_{A}\;\;\int_{A^{\prime }}E_{a_{1},\omega }\left(a_{1}^{\prime }\right)\exp\left[j\omega \tau_{a_{1},a_{1}^{\prime }}\left(r,\omega \right)\right]E_{a_{2},\omega }\left(a_{2}^{\prime }\right)\exp\left[j\omega \tau_{a_{2},a_{2}^{\prime }}\left(r,\omega \right)\right]\;da_{1}\;da_{1}^{\prime }\;da_{2}\;da_{2}^{\prime }\;d\omega$$

Although this does not increase the amount of independent information, this pairwise multiplication artificially increases the number of channels in the summation. The DMAS beamformer assumes that signals with a high degree of coherence should be rewarded by multiplication, whereas incoherent signals should not increase in energy after multiplication.

A fundamental assumption of radar-based imaging is that knowledge of propagation within the breast can be used to synthetically focus the recorded electromagnetic energy to points within the breast. This is represented by the propagation delay, $\tau_{a,a^{\prime }}\left(r,\omega \right)$, in Equation (1). The propagation delay can be calculated from the dielectric properties of the breast, however, in a realistic imaging scenario, the dielectric properties of the breast are not known. Thus, most practical implementations assume a single estimate of the dielectric properties for the entire breast, known as the effective permittivity in this work.

Several patient-specific permittivity estimation methods have been proposed which are reviewed in detail in [24]. Patient-specific permittivity estimation methods based on analyzing images reconstructed within a range of potential permittivity estimates have shown promising results in experimental and clinical test cases [23,26,44,45]. These patient-specific permittivity estimation methods have been shown to improve the sensitivity in 110 experimental test cases including diverse breast phantoms and tumor phantoms [46]. However, in [23,26], the original DAS beamformer was used, and the impact of other beamformers on the method is unknown.

### Evaluation Metrics

To assess the performance of beamformers, it is necessary to identify a set of evaluation metrics for the radar-based images. In many cases, image quality has been defined using the signal-to-mean ratio (SMR) and the signal-to-clutter ratio (SCR). Typically, the SMR is calculated by dividing the mean energy of the tumor area (the area surrounding the maximum intensity of the image) by the mean energy of the image of the ipsilateral breast, where the tumor area is twice the full-width half-maximum (FWHM) of the response of highest energy in the image. Similarly, the SCR is calculated by dividing the maximum energy of the tumor area (i.e., the maximum intensity of the image) by the maximum energy in the background of the image. Additionally, in images of test scenarios with abnormalities, the localization error, or the distance between the maximum response in the image and the location of the abnormality, is used as a measure of quality. Lower localization error means higher image quality.

In this work, the SCR and the FWHM of the images using different beamformers is compared. Although recent studies have suggested that these metrics may not be sufficient to distinguish images of modelled healthy and cancerous cases [47], the SCR and the FWHM are still the standard metrics for image quality quantification.

Additionally, the images are analyzed quantitatively and annotated as containing a tumor or not according to the criteria described in detail in [24]. Broadly speaking, images where the main response has a SCR greater than 1.5 dB are annotated as a positive and considered to be detection. Where the main response is located within the physical extent of the tumor, the image is marked as a true positive, otherwise, it is considered a false positive.

## 3. Experimental Methods

In this section, the experimental methods are described. The BRIGID breast and tumor phantom set was used to assess the performance of the imaging algorithms which is fully presented in [48]. The breast and tumor phantoms are fabricated from a polyurethane mixture combined with graphite and carbon black powders to control the dielectric properties [49]. Once cured, the mixture is solid, and can be used to create a modular and stable test platform [48,50].

Breast phantoms were hemispherical with diameter of 14 cm and consisted of three tissue types:

- a hemispherical skin which varied between 1 to 3 mm in thickness with relative permittivity of $\varepsilon_{r}\approx 30$ and electrical conductivity of $\sigma \approx 2\;S/m^{-1}$ at 3 GHz;
- conical glandular structures to model breast lobes radiating from the areola with relative permittivity of $\varepsilon_{r}\approx 45$ and electrical conductivity of $\sigma \approx 2.5\;S/m^{-1}$ at 3 GHz;
- an adipose layer with relative permittivity of $\varepsilon_{r}\approx 8$ and electrical conductivity of $\sigma \approx 0.1\;S/m^{-1}$.

In each breast phantom, a hole was left in which a tumor “plug” could be inserted. Each tumor phantom was embedded in a plug of adipose tissue, and the tumor phantom had a mean relative permittivity of $\varepsilon_{r}\approx 70$ and mean electrical conductivity of $\sigma \approx 6\;S/m^{-1}$. The contrast between the dielectric properties of tumor and glandular tissues in these breast phantoms is similar to the contrast between healthy and glandular tissues in the literature [1,51,52,53].

The BRIGID phantom set contains breast phantoms which vary according to the volume of glandular tissue present within the phantom, known as the volume glandular fraction (VGF). Recent advances in three-dimensional imaging using breast tomosynthesis have allowed the VGF to be quantified, such as in a study of 219 women in [54]. This study suggests that breast density in terms of VGF may be overestimated from the two-dimensional slices used in X-ray mammography, and that the average VGF of breasts even in BI-RADS Class D maybe be as low as 20%.

**Figure 1:**
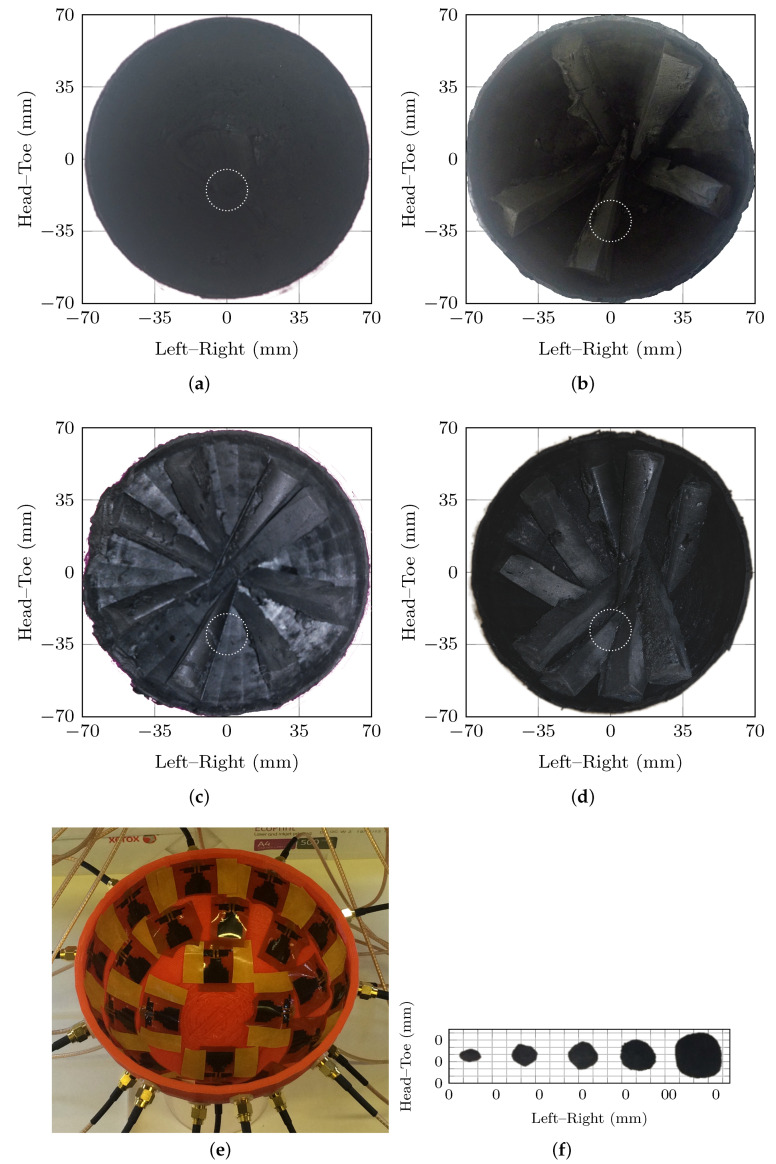
The four breast phantoms are shown in (**a**–**d**) containing 0%, 10%, 15% and 20% VGF, respectively. The tumor location is shown by the dashed, white circle. The 24 flexible microstrip antennas shown housed in the radome are shown in (**e**) and the 5 tumour phantoms are showin in (**f**).

In this work, images of four breast phantoms with VGF varying from 0% to 20% are analyzed as the healthy cases, specifically 0%, 10%, 15% and 20% as shown in Figure 1. These breast phantoms are representative of the variation in VGF present in the population in the sense that breast phantoms with VGF covering more than 80% of the population are included [54]. Each of the four breast phantoms can also be combined with a tumor phantom to model a cancerous case, the location of the tumor phantom is shown in Figure 1 by the dashed white circle. Five spherical tumor phantoms ranging in diameter from 5 mm to 20 mm are used, representing plugs 1 to 5 from [48], which are shown in Figure 1f. In total, there are 20 test cases with tumor phantoms (five tumor phantoms in four breast phantoms) and four test cases without tumors (four breast phantoms with an adipose tissue in place of the tumor phantom).

Signals are acquired from the breast phantoms using experimental hardware developed at NUI Galway, Ireland which is described fully in [23]. Experimental data between 2 GHz and 4 GHz were acquired using a ZNB40 2-port VNA and ZN-Z84 24-port switching matrix (Rohde & Schwartz GmbH, Munich, Germany) in the frequency domain. 24 flexible microstrip antennas were used to collect the multistatic scan. The antennas were first developed and used at McGill University in experimental and healthy clinical case studies [55,56]. The antennas housed in the radome are shown in Figure 1e. The antennas were designed to be in contact with a material with relative permittivity close to skin and were in direct contact with the skin of the phantom in this system. The imaging system was calibrated to the antenna using open/short/load and through measurements for all channels.

A reference scan with a homogeneous breast phantom was used to account for differences between the antennas. This reference scan was used to calculate a “calibration factor” which was then applied to neighboring antennas such that the response of each antenna was the same. While effective in these experimental studies, this calibration is not optimized for use with patients without optimization to ensure the breast contacts the antennas correctly. Many approaches have been proposed in the literature to overcome these issues:

- inclusion of a suction system to ensure the breast contacts the radome [57];
- undersizing the radome compared to the reported breast size to improve the fit [56];
- use of a coupling medium to help reduce air gaps between the breast and the antennas [2,56,58];
- automated metrics to ensure the breast is well-situated prior to the scan [58].

After acquisition, all signal processing and imaging was performed in the frequency domain using MERIT, an open-source toolbox for microwave imaging [15]. First, rotational subtraction was performed to remove the large skin reflection and other artefacts [23]. In this experimental set-up, a rotational angle of 36deg was used corresponding to the angle between neighboring antennas. Although rotational subtraction has also been used with the MARIA^®^ system and hundreds of women [2,58], the optimal angle of rotation and the impact of rotational subtraction on image quality are not well studied.

Next, an assumed estimate of the relative permittivity of the breast was used to synthetically focus all signals to a point of interest within the breast [23]. Considering the generic radar-based beamformer shown in Equation (1), this is discretized and simplified to become the DAS beamformer in the frequency domain:

(3) $$I\left(r,\varepsilon_{r}^{\prime }\right)=\sum_{A}\sum_{A^{\prime }}E_{a,a^{\prime }}^{\prime }\left(r\right)\exp\left[\frac{j\omega }{c_{0}}\sqrt{\varepsilon_{r}^{\prime }}\left(∥a-r∥+∥a^{\prime }-r∥\right)\right]$$

where $I(r,\varepsilon_{r}^{\prime })$ is the beamformer with the average permittivity of the breast, $\varepsilon_{r}^{\prime }$, as a parameter; the antenna locations are the 24 antenna locations shown in Figure 1e; and the propagation paths are assumed to be the straight-line paths between the antennas and the points of interest. The DMAS beamformer is implemented similarly; however, prior to the beamformer being applied, the signals are pairwise multiplied.

For each test case, a cost function is used to estimate the “quality” of that image. Specifically, the Absolute Gradient, $\Phi_{\mathrm{DMA}}^{G}$, from [23] is applied. For each set of artefact-removed signals corresponding to a single test case, 50 images are reconstructed: specifically, both DAS and DMAS images are reconstructed at 25 permittivity values between $\varepsilon_{r}^{\prime }=8$ and $\varepsilon_{r}^{\prime }=14$. For each of the 24 test cases (5 tumor phantoms in 4 breast phantoms and the 4 breast phantoms without tumor phantoms), the 50 images per case are analyzed qualitatively and quantitavely below.

## 4. Results

The results of this paper are presented in a three stages:

1. first, the impact of permittivity estimation in phantoms without abnormalities is discussed in Section 4.1, considering both beamformers and the four breast phantoms;
2. secondly, the ability of DMAS to compensate for errors in the permittivity estimation process is considered in Section 4.2;
3. thirdly, the permittivity estimation algorithms are applied to images of the same scene reconstructed with different beamformers to estimate if the characteristics of the images are different in Section 4.3.

### 4.1. Algorithm Performance in Test Cases without Abnormalities

**Figure 2:**
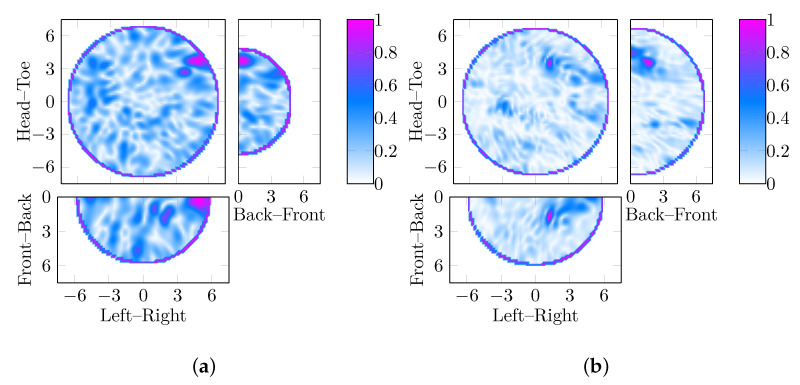

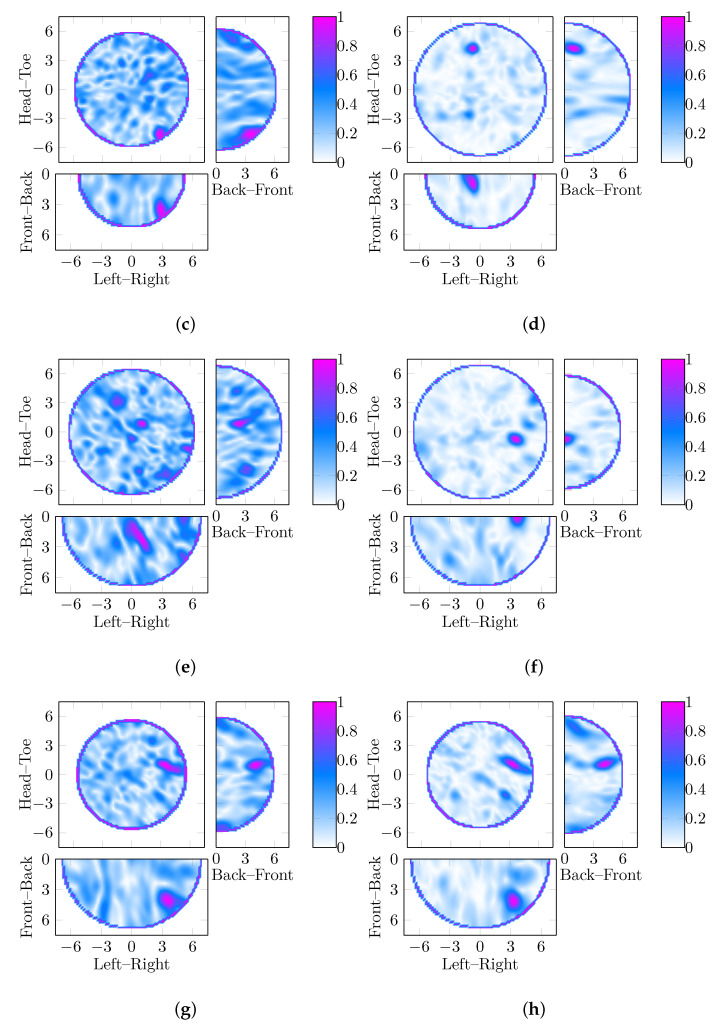
Images using DAS and DMAS of four breast phantoms with between 0% and 20% VGF. The images reconstructed using DAS are shown in the leftmost column (**a**, **c** and **e**) in order of increasing VGF (10%, 15% and 20% respectively). The corresponding image of the same test case reconstructed using DMAS is shown in the rightmost column (**b**, **d** and **f**). As a response that is very close to the skin would likely be regarded as an artefact, in the less dense breast phantoms, (**a**,**c**) reconstructed with DAS could be considered to be no detection, whereas the DMAS for the same breast phantom shows a response which is more in the center of the breast and more likely to be interpreted as a false positive. All dimensions are in cm, and the three slices shown (coronal, sagittal and axial) are cross-sections at the location of the maximum amplitude of the image in each case.

In this section, the impact of permittivity estimation on beamformer selection in breast phantoms without abnormalities is discussed. First, the images of the four breast phantoms without abnormalities using both the DAS and the DMAS beamformers are investigated in Figure 2. These images are reconstructed at $\varepsilon_{r}=8.75$, which is the optimal permittivity based on maximizing the sensitivity for all test cases with abnormalities. Figure 2a,c,e,g are reconstructed with DAS, whereas Figure 2b,d,f,h are reconstructed with DMAS.

The images in Figure 2 highlight the potential challenges in distinguishing scenarios containing tumor phantoms from scenarios without tumor phantoms. Due to the reflections from other tissue boundaries within the breast (i.e., between the skin and the adipose tissue not fully removed by artefact removal and between the glandular structures and the adipose tissue), images of breast phantoms without tumor phantoms can have characteristics similar to images of tumor phantoms embedded in breast phantoms.

Considering the least dense breast phantom initially (Figure 2a,b), it can be seen that the DMAS image shows a distinct response in the middle of the breast whereas the DAS image of the same scene does not. A similar result is visible in Figure 2c,d where the DMAS image shows a more distinct response than the DAS image. These images suggest that DMAS may make the specificity of radar-based imaging worse even in breast phantoms with minimal dielectric contrast in the breast phantom interior.

However, in the denser breast phantoms, Figure 2e,f, both DAS and DMAS images show responses, as is expected as the fibroglandular structures have significant dielectric contrast to the adipose background. Interestingly, in the case of the 15% VGF breast phantom in Figure 2e,f, Where, as the DAS image highlights a response in the middle of the breast, the DMAS image highlights a response very close to the chest wall for the same scene. For the densest breast phantom in Figure 2g,h, it can be seen that the DMAS image shows a response in the same location as the DAS image, but with higher SCR.

Considering the images selected by using the permittivity estimation method described in [23], the parameter search algorithm selects $\varepsilon_{r}=8$ for images of the 15% and 20% breast phantoms for both the DAS and DMAS images. In both cases, the responses are in the same location (within 5 mm) and could both be interpreted as containing a response that could be a tumor. However, the DMAS images have SCR of 2.62 dB and 3.06 dB for the 15% and 20% breast phantoms respectively, compared to 0.56 dB and 0.29 dB for the DAS images. These are shown quantitively in Table 1.

For the less dense breast phantoms, the parameter search algorithm would select images reconstructed at different permittivity values, $\varepsilon_{r}=10.5$ and $\varepsilon_{r}=9$ for DAS and DMAS respectively for the 0% breast phantom and $\varepsilon_{r}=8.75$ and $\varepsilon_{r}=8$ for the 10% breast phantom. No clear trend is visible: for 0% VGF the DAS image selected by the parameter search algorithm has higher SCR compared to the DMAS image selected by the parameter search algorithm (2.6 dB compared to 1.8 dB), whereas the reverse is true for 10% (1.6 dB compared to 2.0 dB). However, even in the simple and limited case studies shown in this section, the choice of beamformer may impact the specificity, suggesting that this should be evaluated in all beamformer comparison studies.

### 4.2. Effects of Permittivity Estimation on Performance

In this section, the effect of using DMAS instead of DAS are analyzed. The reconstruction permittivities are chosen using the parameter search algorithm applied to the DAS images. Additionally, case studies are presented which show how the beamformer comparison can depend on the permittivity chosen for reconstruction.

**Figure 3:**
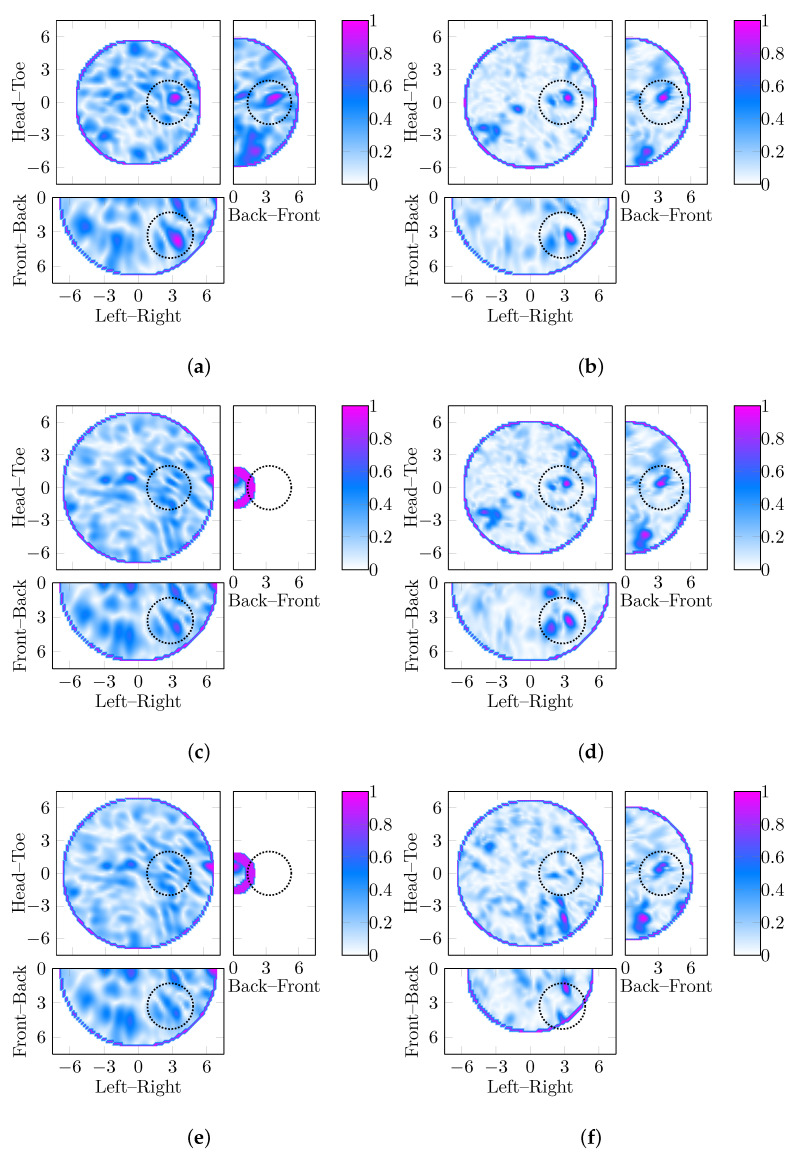
Six images of a 2 cm diameter tumor in the breast phantom with 20% VGF are shown, reconstructed with the DAS and DMAS beamformers are three different reconstruction permittivities. The DAS images are shown in the leftmost column in order of increasing permittivity, similarly, the corresponding DMAS images are shown in the rightmost column. Depending on the reconstruction permittivity chosen between $\varepsilon_{r}=11.75$ and $\varepsilon_{r}=12.75$, both beamformers perform the same and correctly localize the tumor in (**a**) and (**b**), DMAS outperforms DAS in (**c**) and (**d**), or neither DAS nor DMAS localize the tumor in (**e**) and (**f**). All dimensions are in cm, and the three slices shown (coronal, sagittal and axial) are cross-sections at the location of the maximum amplitude of the image in each case.

First, a case study is shown in Figure 3 where all six images are reconstructed from the same experimental case but using a reconstruction permittivity estimate of $\varepsilon_{r}=11.75$ in Figure 3a,b, $\varepsilon_{r}=12.25$ in Figure 3c,d, and $\varepsilon_{r}=12.75$ in Figure 3e,f. Figure 3a,c,e are reconstructed with DAS, whereas Figure 3b,d,f are reconstructed with DMAS.

Considering the lowest estimate of $\varepsilon_{r}=11.75$ in Figure 3a,b, the strongest response is correctly located in the images reconstructed with both DAS and DMAS. The FWHM is similar in both cases (approximately 8 mm), but the SCR of the image reconstructed with DMAS is 1.08 dB whereas the SCR of the image reconstructed with DAS is 0.09 dB.

Overestimating the relative permittivity by 5% in Figure 3c,d, the image reconstructed with DAS does not identify the tumor in the correct location (a false negative) whereas the image reconstructed with DMAS does correspond with the tumor location, albeit with a lower SCR of 0.42 dB. This result may indicate that improving the beamforming algorithm can compensate for errors in the relative permittivity estimates for image reconstruction.

Finally, overestimating the relative permittivity by 10% in Figure 3e,f, neither the image reconstructed with DAS nor the image reconstructed with DMAS show a response in the correct location. However, whereas the image reconstructed with DAS would be regarded as a false negative, the image reconstructed with DMAS does have a response in the image, but in the wrong location.

Considering all 20 cases (five tumor phantoms different in size in four breast phantoms differing in VGF), 14 are detected using DAS and permittivity estimation. For these 14, the DAS image is compared to the DMAS image at the same estimate of the permittivity. For 3 of the 14 images (P5 in 20%; and P3 and P4 in 15%), the DMAS image is “sharper” with SCR at least 1 dB higher and FWHM approximately 2 mm smaller. For a further 6 of the 14 (all from the 0% and 10% phantoms), the images were similar where the SCR was within 1 dB and the localization was within 1 cm. However, for 5 of the 14 cases (P1 from 0%; P1 from 10%; P2 and P5 from 15%; and P4 from 20%), the image deteriorated such that the tumor was not detected: for all five, the location of the maximum image response was more than 5 cm away from the location of the tumor phantom.

For the 6 of 20 cases where DAS and permittivity estimation did not detect the tumor, in 2 cases, the DMAS image was very similar to the DAS image but with less clutter. In 3 more cases, the DMAS image would also not have located the tumor in the right location, but the main response in the image was in a different location when compared to the DAS image. In only 1 case, P5 in 10% phantom, did the DMAS image reconstructed at the same permittivity as the DAS image correctly identify the tumor where the DAS image would not have.

### 4.3. Parameter Search Performance Using both Beamformers

Finally, in this section, images selected by DMAS and the permittivity estimation algorithm are compared to the DAS image at the same permittivity as the DMAS images, and to the DAS images selected by the permittivity estimation algorithm. Overall, the sensitivity from the DMAS images with permittivity estimation was 10 out of 20 cases compared to the 14 out of 20 for DAS with permittivity estimation.

For the 20 breast and tumor phantom combinations, in 9 cases the permittivity estimation algorithm selected a DMAS image reconstructed within $\Delta \varepsilon_{r}=0.5$ of the DAS image. Of these 9 cases, 7 of the DAS images correctly identified the tumor in the correct location where as 6 of the DMAS images did. The majority of these nine cases were in the lower density phantoms: 6 of the 9 were images of phantoms with 0% and 10% VGF. This is expected, as images are less sensitive to the reconstruction permittivity at lower VGF.

For the remaining 11 cases where the reconstruction permittivity selected by the parameter search algorithm from the DMAS image differed from the DAS image, 7 of the 11 DAS images correctly identified the tumor whereas only 3 of the 11 DMAS images did. Interestingly, of the four images where DAS with permittivity estimation outperformed DMAS with permittivity estimation, two (P1 and P5) were in the breast phantom with 0% VGF.

## 5. Conclusions

In this work, the potential impact of permittivity estimation on beamformer comparative study design is highlighted. Although many comparative studies of various beamformers have been published in the literature, most use the simplifying assumption that the same estimate of the permittivity can be used for all test cases, although this is known to impair the image quality. Similarly, the majority include only test cases with tumors, and do not compare the beamformers in test cases without tumors.

The original DAS beamformer is compared to the DMAS beamformer, which was identified as a promising beamformer from a recent comparative study using five clinical case studies. The results in this paper suggest that DMAS may reconstruct an image of higher quality (defined primarily in terms of SCR) than the original DAS, however, this may result in more false positives such as in the less dense breast phantoms in this paper.

The importance of including realistic patient-specific estimation when comparing beamformers is also shown. A case study showing how the conclusion of a beamformer comparison study may vary depending on the permittivity estimate is presented. In this case study, as the estimate varies by 5%, it could be concluded that both beamformers perform the same, or that DMAS localizes the tumor where DAS does not, or that neither DAS nor DMAS localizes the tumor. Hence it is important to consider realistic and varied test cases when comparing beamformer performance.

Future work in this area should include the development of metrics to quantify the image quality which are useful to distinguish images of healthy and abnormal breasts. Future studies should consider the effects of permittivity estimation when designing and comparing beamformer performance experimentally and clinically.

## Figures and Tables

**Table 1:** The SCR and FWHM of images of breast phantoms without abnormalities containing 0% and 10% VGF, reconstructed with DAS and DMAS and a parameter search algorithm. For 0% VGF: $\varepsilon_{r}^{\mathrm{DAS}}=10.5$ and $\varepsilon_{r}^{\mathrm{DMAS}}=9$ whereas for 10% VGF, $\varepsilon_{r}^{\mathrm{DAS}}=8.75$ and $\varepsilon_{r}^{\mathrm{DMAS}}=8$.

	SCR (dB)				FWHM (mm)			
	0% VGF		10% VGF		0% VGF		10% VGF	
	$\varepsilon_{r}^{\mathrm{DAS}}$	$\varepsilon_{r}^{\mathrm{DMAS}}$	$\varepsilon_{r}^{\mathrm{DAS}}$	$\varepsilon_{r}^{\mathrm{DMAS}}$	$\varepsilon_{r}^{\mathrm{DAS}}$	$\varepsilon_{r}^{\mathrm{DMAS}}$	$\varepsilon_{r}^{\mathrm{DAS}}$	$\varepsilon_{r}^{\mathrm{DMAS}}$
DAS	2.6	1.5	1.6	0.75	10	12	13	7
DMAS	0.7	1.8	2.6	2.0	7	6	9	10

## References

[1] O’Loughlin D., O’Halloran M., Moloney B.M., Glavin M., Jones E., Elahi M.A. (2018). Microwave Breast Imaging: Clinical Advances and Remaining Challenges. Trans. Biomed. Eng..

[2] Preece A.W., Craddock I.J., Shere M., Jones L., Winton H.L. (2016). MARIA M4: Clinical Evaluation of a Prototype Ultrawideband Radar Scanner for Breast Cancer Detection. J. Med. Imaging.

[3] Porter E., Coates M., Popović M. (2016). An Early Clinical Study of Time-Domain Microwave Radar for Breast Health Monitoring. IEEE Trans. Biomed. Eng..

[4] Song H., Sasada S., Kadoya T., Okada M., Arihiro K., Xiao X., Kikkawa T. (2017). Detectability of Breast Tumor by a Hand-Held Impulse-Radar Detector: Performance Evaluation and Pilot Clinical Study. Sci. Rep..

[5] Yang F., Sun L., Hu Z., Wang H., Pan D., Wu R., Zhang X., Chen Y., Zhang Q. (2017). A Large-Scale Clinical Trial of Radar-Based Microwave Breast Imaging for Asian Women: Phase I. Proceedings of the International Symposium on Antennas and Propagation (APSURSI).

[6] Song H., Sasada S., Masumoto N., Kadoya T., Shiroma N., Orita M., Arihiro K., Okada M., Kikkawa T. (2018). Detectability of Breast Tumors in Excised Breast Tissues of Total Mastectomy by IR-UWB-Radar-Based Breast Cancer Imaging. IEEE Trans. Biomed. Eng..

[7] Wörtge D., Moll J., Krozer V., Bazrafshan B., Hübner F., Park C., Vogl T. (2018). Comparison of X-ray Mammography and Planar UWB Microwave Imaging of the Breast: First Results from a Patient Study. Diagnostics.

[8] Fasoula A., Anwar S., Toutain Y., Duchesne L. (2017). Microwave Vision: From RF Safety to Medical Imaging. Proceedings of the 11th European Conference on Antennas and Propagation (EuCAP).

[9] Fasoula A., Duchesne L., Gil Cano J., Lawrence P., Robin G., Bernard J.G. (2018). On-Site Validation of a Microwave Breast Imaging System, before First Patient Study. Diagnostics.

[10] Duchesne L., Fasoula A., Kaverine E., Robin G., Bernard J.G. Wavelia Microwave Breast Imaging: Identification and Mitigation of Possible Sources of Measurement Uncertainty. Proceedings of the 13th European Conference on Antennas and Propagation (EuCAP).

[11] Bolomey J.C., Lakhtakia A., Furse C.M. (2018). Crossed Viewpoints on Microwave-Based Imaging for Medical Diagnosis: From Genesis to Earliest Clinical Outcomes. The World of Applied Electromagnetics.

[12] Nikolova N.K. (2014). Microwave Biomedical Imaging. Wiley Encyclopedia of Electric and Electronics Engineering.

[13] Conceição R.C., Mohr J.J., O’Halloran M. (2016). An Introduction to Microwave Imaging for Breast Cancer Detection.

[14] Nikolova N.K. (2017). Introduction to Microwave Imaging.

[15] O’Loughlin D., Elahi M.A., Porter E., Shahzad A., Oliveira B.L., Glavin M., Jones E., O’Halloran M. Open-Source Software for Microwave Radar-Based Image Reconstruction. Proceedings of the 12th European Conference on Antennas and Propagation (EuCAP).

[16] Klemm M., Craddock I.J., Leendertz J.A., Preece A.W., Benjamin R. (2009). Radar-Based Breast Cancer Detection Using a Hemispherical Antenna Array—Experimental Results. IEEE Trans. Antennas Propag..

[17] Xie Y., Guo B., Xu L., Li J., Stoica P. (2006). Multistatic Adaptive Microwave Imaging for Early Breast Cancer Detection. IEEE Trans. Biomed. Eng..

[18] O’Halloran M., Glavin M., Jones E. (2009). Effects of Fibroglandular Tissue Distribution on Data-Independent Beamforming Algorithms. Prog. Electromagn. Res..

[19] Byrne D., O’Halloran M., Glavin M., Jones E. (2010). Data Independent Radar Beamforming Algorithms for Breast Cancer Detection. Prog. Electromagn. Res..

[20] Moll J., Kexel C., Krozer V. A Comparison of Beamforming Methods for Microwave Breast Cancer Detection in Homogeneous and Heterogeneous Tissue. Proceedings of the Microwave Conference (EuMC).

[21] Elahi M.A., Lavoie B.R., Porter E., Glavin M., Jones E., Fear E.C., O’Halloran M. (2017). Comparison of Radar-Based Microwave Imaging Algorithms Applied to Experimental Breast Phantoms. Proceedings of the 32nd International Union of Radio Science (URSI) General Assembly and Scientific Symposium.

[22] Elahi M.A., O’Loughlin D., Lavoie B.R., Glavin M., Jones E., Fear E.C., O’Halloran M. (2018). Evaluation of Image Reconstruction Algorithms for Confocal Microwave Imaging: Application to Patient Data. Sensors.

[23] O’Loughlin D., Oliveira B.L., Elahi M.A., Glavin M., Jones E., Popović M., O’Halloran M. (2017). Parameter Search Algorithms for Microwave Radar-Based Breast Imaging: Focal Quality Metrics as Fitness Functions. Sensors.

[24] O’Loughlin D., Oliveira B.L., Santorelli A., Porter E., Glavin M., Jones E., Popović M., O’Halloran M. (2019). Sensitivity and Specificity Estimation Using Patient-Specific Microwave Imaging in Diverse Experimental Breast Phantoms. IEEE Trans. Med. Imaging.

[25] O’Loughlin D., Krewer F., Glavin M., Jones E., O’Halloran M. (2017). Focal Quality Metrics for the Objective Evaluation of Confocal Microwave Images. Int. J. Microw. Wirel. Technol..

[26] Lavoie B.R., Okoniewski M., Fear E.C. (2016). Estimating the Effective Permittivity for Reconstructing Accurate Microwave-Radar Images. PLoS ONE.

[27] O’Loughlin D., Glavin M., Jones E., O’Halloran M. (2018). Evaluation of Experimental Microwave Radar-Based Images: Evaluation Criteria. Proceedings of the Antennas and Propagation Society International Symposium (APSURSI).

[28] Iskander M.F., Durney C.H. (1980). Electromagnetic Techniques for Medical Diagnosis: A Review. Proc. IEEE.

[29] Larsen L., Jacobi J. (1982). Microwaves Offer Promise as Imaging Modality. Diagn. Imaging.

[30] Fear E.C., Hagness S.C., Meaney P.M., Okoniewski M., Stuchly M.A. (2002). Enhancing Breast Tumor Detection with Near-Field Imaging. IEEE Microw. Mag..

[31] Fear E.C. (2005). Microwave Imaging of the Breast. Technol. Cancer Res. Treat..

[32] Nikolova N.K. (2011). Microwave Imaging for Breast Cancer. IEEE Microw. Mag..

[33] Meaney P.M. (2013). Microwave Imaging: Perception and Reality. Expert Rev. Med. Devices.

[34] Crocco L. (2015). Microwaves for Medical Imaging: Some Possible Pathways for an Accelerated Progress towards Clinical Practice. New Horizons Transl. Med..

[35] Chandra R., Zhou H., Balasingham I., Narayanan R.M. (2015). On the Opportunities and Challenges in Microwave Medical Sensing and Imaging. IEEE Trans. Biomed. Eng..

[36] Kwon S., Lee S. (2016). Recent Advances in Microwave Imaging for Breast Cancer Detection. Int. J. Biomed. Imaging.

[37] Modiri A., Goudreau S., Rahimi A., Kiasaleh K. (2017). Review of Breast Screening: Towards Clinical Realization of Microwave Imaging. Med. Phys..

[38] Li X., Hagness S.C. (2001). A Confocal Microwave Imaging Algorithm for Breast Cancer Detection. Microw. Wirel. Components Lett. IEEE.

[39] Nilavalan R., Gbedemah A., Craddock I.J., Li X., Hagness S.C. (2003). Numerical Investigation of Breast Tumour Detection Using Multi-Static Radar. Electron. Lett..

[40] Fear E.C., Bourqui J., Curtis C.F., Mew D., Docktor B., Romano C. (2013). Microwave Breast Imaging With a Monostatic Radar-Based System: A Study of Application to Patients. IEEE Trans. Microw. Theory Tech..

[41] Shao W., Edalati A., McCollough T.R., McCollough W.J. (2018). A Time-Domain Measurement System for UWB Microwave Imaging. IEEE Trans. Microw. Theory Tech..

[42] Islam M., Samsuzzaman M., Islam M., Kibria S. (2018). Experimental Breast Phantom Imaging with Metamaterial-Inspired Nine-Antenna Sensor Array. Sensors.

[43] Lim H.B., Nhung N.T.T., Li E.P., Thang N.D. (2008). Confocal Microwave Imaging for Breast Cancer Detection: Delay-Multiply-and-Sum Image Reconstruction Algorithm. IEEE Trans. Biomed. Eng..

[44] O’Loughlin D., Krewer F., Glavin M., Jones E., O’Halloran M. (2016). Estimating Average Dielectric Properties for Microwave Breast Imaging Using Focal Quality Metrics. Proceedings of the 10th European Conference on Antennas and Propagation (EuCAP).

[45] O’Loughlin D., Glavin M., Jones E., O’Halloran M. (2016). Optimisation of Confocal Microwave Breast Images Using Image Focal Metrics. Proceedings of the 22nd Bioengineering in Ireland (BINI).

[46] O’Loughlin D., Oliveira B.L., Glavin M., Jones E., O’Halloran M. (2019). Advantages and Disadvantages of Parameter Search Algorithms for Permittivity Estimation for Microwave Breast Imaging. Proceedings of the 13th European Conference on Antennas and Propagation (EuCAP).

[47] O’Loughlin D., Oliveira B.L., Glavin M., Jones E., O’Halloran M. (2018). Effects of Interpatient Variance on Microwave Breast Images: Experimental Evaluation. Proceedings of the 40th Annual International Conference of the Engineering in Medicine and Biology Society (EMBC).

[48] Oliveira B.L., O’Loughlin D., O’Halloran M., Porter E., Glavin M., Jones E. (2018). Microwave Breast Imaging: Experimental Tumour Phantoms for the Evaluation of New Breast Cancer Diagnosis Systems. Biomed. Phys. Eng. Express.

[49] Garrett J., Fear E.C. (2015). A New Breast Phantom With a Durable Skin Layer for Microwave Breast Imaging. IEEE Trans. Antennas Propag..

[50] Santorelli A., Laforest O., Porter E., Popović M. (2015). Image Classification for a Time-Domain Microwave Radar System: Experiments with Stable Modular Breast Phantoms. Proceedings of the 9th European Conference on Antennas and Propagation (EuCAP).

[51] Lazebnik M., McCartney L., Popović D., Watkins C.B., Lindstrom M.J., Harter J., Sewall S., Magliocco A., Booske J.H., Okoniewski M. (2007). A Large-Scale Study of the Ultrawideband Microwave Dielectric Properties of Normal Breast Tissue Obtained from Reduction Surgeries. Phys. Med. Biol..

[52] Lazebnik M., Popović D., McCartney L., Watkins C.B., Lindstrom M.J., Harter J., Sewall S., Ogilvie T., Magliocco A., Breslin T.M. (2007). A Large-Scale Study of the Ultrawideband Microwave Dielectric Properties of Normal, Benign and Malignant Breast Tissues Obtained from Cancer Surgeries. Phys. Med. Biol..

[53] Sugitani T., Kubota S., Kuroki S., Sogo K., Arihiro K., Okada M., Kadoya T., Hide M., Oda M., Kikkawa T. (2014). Complex Permittivities of Breast Tumor Tissues Obtained from Cancer Surgeries. Appl. Phys. Lett..

[54] Huang S.Y., Boone J.M., Yang K., Packard N.J., McKenney S.E., Prionas N.D., Lindfors K.K., Yaffe M.J. (2011). The Characterization of Breast Anatomical Metrics Using Dedicated Breast CT. Med. Phys..

[55] Bahramiabarghouei H., Porter E., Santorelli A., Gosselin B., Popović M., Rusch L.A. (2015). Flexible 16 Antenna Array for Microwave Breast Cancer Detection. IEEE Trans. Biomed. Eng..

[56] Porter E., Bahrami H., Santorelli A., Gosselin B., Rusch L.A., Popović M. (2016). A Wearable Microwave Antenna Array for Time-Domain Breast Tumor Screening. IEEE Trans. Med. Imaging.

[57] Kuwahara Y., Malik A.M. (2017). Microwave Imaging for Early Breast Cancer Detection. New Perspectives in Breast Imaging.

[58] Shere M., Lyburn I., Sidebottom R., Massey H., Gillett C., Jones L. (2019). MARIA^®^ M5: A Multicentre Clinical Study to Evaluate the Ability of the Micrima Radio-Wave Radar Breast Imaging System (MARIA^®^) to Detect Lesions in the Symptomatic Breast. Eur. J. Radiol..

